# Assessment of tissue homogenate levels of TGM1, PPL and KRT8 in a group of patients with HNSCC tumors and matched surgical margin samples

**DOI:** 10.3389/fonc.2026.1694449

**Published:** 2026-01-30

**Authors:** Dariusz Nałęcz, Agata Świętek, Dorota Hudy, Zofia Złotopolska, Jakub Opyrchał, Radosław Lenckowski, Michał Dawidek, David Aebisher, Joanna Katarzyna Strzelczyk

**Affiliations:** 1 Department of Otolaryngology and Maxillofacial Surgery, St. Vincent De Paul Hospital, Gdynia, Poland; 2 Department of Medical and Molecular Biology, Faculty of Medical Sciences in Zabrze, Medical University of Silesia in Katowice, Zabrze, Poland; 3 Silesia LabMed Research and Implementation Center, Medical University of Silesia in Katowice, Zabrze, Poland; 4 2nd Department of Oncologic Surgery, Maria Skłodowska-Curie National Research Institute of Oncology, Gliwice, Poland; 5 Department of Pathomorphology, Gdynia Center of Oncology, Pomeranian Hospitals, Gdynia, Poland; 6 Department of Head and Neck Reconstructive Surgery and Robotic Surgery, Gdynia, Poland; 7 Department of Photomedicine and Physical Chemistry, Faculty of Medicine, Collegium Medium, University od Rzeszów, Rzeszów, Poland

**Keywords:** head and neck squamous cell carcinoma, transglutaminase 1, periplakin, keratin 8, protein level

## Abstract

**Introduction:**

Head and neck squamous cell carcinoma (HNSCC) is a group of malignancies with significantly increasing incidence and mortality. Associated TGM1, PPL, and KRT proteins are involved in epithelial cell structure, adhesion, and differentiation.

**Objective, patients and methods:**

This study aimed to evaluate TGM1, PPL, and KRT8 levels in tumors and matched surgical margin samples from 52 HNSCC patients and assess correlations with clinical and demographic variables using ELISA.

**Results:**

No significant differences in TGM1, PPL, and KRT8 levels were found between tumor and margin samples. However, in tumor tissue, TGM1 and KRT8 levels showed a statistically significant association with T status. In margins, PPL and KRT8 levels were also associated with T status. Additionally, PPL and TGM1 levels were correlated with N status in both tumor and margin samples, respectively. A significantly higher level of PPL was observed in OSCC tumors compared to HPSCC+LSCC. TGM1 levels in tumor and margin samples were correlated in patients with concomitant diseases. Analysis of HPV and p16 status revealed differences in PPL and KRT8 levels between tumor and margin samples. Furthermore, differences in PPL, TGM1, and KRT8 levels were observed in relation to smoking and alcohol use, distinguishing regular or occasional users from abstinent patients.

**Conclusions:**

Our results suggest that impaired TGM1, PPL, and KRT8 signaling pathways might play a role in HNSCC, indicating their potential relevance for future diagnostic and therapeutic investigations. Further studies are needed to confirm our findings, clarify the mechanistic role of these proteins in disease progression, and assess their clinical utility.

## Introduction

Head and neck squamous cell carcinoma (HNSCC) comprises a heterogeneous group of malignancies in the upper respiratory and gastrointestinal tracts (1). Globally, HNSCC incidence and mortality have increased significantly, with an estimated 500,000 new cases annually (2). Major risk factors include tobacco, alcohol, poor oral hygiene, missing teeth, dental caries, and untreated periodontal disease (3). In recent years, viral infections, particularly HPV, have gained prominence in the etiopathogenesis of HNSCC, especially in oral squamous cell carcinomas (OSCC) and oropharyngeal carcinomas (OPSCC) (4). HNSCCs also show diverse molecular abnormalities, and advances in molecular biology have improved our understanding of the pathways involved. Despite progress in diagnosis and treatment, the 5-year progression-free survival remains at 40–50%, with limited improvement in metastatic cases (5). This is likely due to the absence of effective screening tools and diagnostic biomarkers, leading to late-stage diagnoses. Biomarkers, classified as diagnostic, prognostic, or predictive, are crucial for early detection, disease monitoring, and therapy response. Although many potential biomarkers have been proposed, few have reached clinical implementation (6).

Transglutaminase 1 (TGM1) belongs to the transglutaminase enzyme group and is involved in the processes of forming the keratinized cell envelope, which is important for maintaining the dermal barrier against physical, chemical, and microbiological agents. TGM1 forms strong crosslinks between structural proteins that form the keratinized cell envelope, providing strength and stability to the epidermis (7). Although dysregulated transglutaminase activity has been described in several malignancies (8, 9), the specific role of TGM1 in HNSCC remains largely unknown. Considering that HNSCC arises from highly differentiated squamous epithelium and often exhibits profound disturbances in keratinocyte maturation and barrier integrity, the contribution of TGM1 to tumor biology represents a notable gap in our current knowledge.

Periplakin (PPL) is a member of the plakin family of cytolytic proteins and is a major component of the precursor of the cornified envelope found beneath the plasma membrane of differentiating keratinocytes. Furthermore, PPL is also found in the desmosomes of keratinocytes, other epithelial cells, and tissues exposed to mechanical dynamics, such as the heart, lung, and skeletal muscle (10). PPL interacts with other proteins, taking part in the regulation of signaling pathways modulating cell adhesion, migration, and differentiation processes, among others. In turn, abnormalities in the proteins of the plakin family are associated with numerous abnormalities in tissue integrity, the stability of the keratinized epidermal envelope of the skin, and the functioning of the nervous and muscular systems (11). Importantly, it has been shown that PPL, by regulating invasion, proliferation, metastasis, and immunity processes, can correlate with the development and progression of several types of cancer, including bladder cancer, triple negative breast cancer, colon cancer, and esophageal cancer (12). The involvement of PPL in HNSCC has not been systematically assessed. Given the central role of desmosomal remodeling and loss of epithelial cohesion in HNSCC pathogenesis, the lack of data on PPL represents a clear research gap that warrants investigation.

Keratin 8 (KRT8) is a member of the type II keratin family and serves as a major component of the cytoskeleton. Together with KRT18, it forms intermediate filaments in the cytoplasm of monolayers of epithelial cells (13). KRT8 is involved in the processes of maintaining the structural integrity of cells and in signal transduction and cell differentiation (14). Abnormalities in KRT8 expression may be associated with the occurrence of various types of cancer (15). In addition, some studies have shown the likely involvement of KRT8 in key tumor processes such as cell migration, adhesion and drug resistance (16). Studies specifically addressing KRT8 in HNSCC are scarce. This is noteworthy because cytoskeletal reprogramming and partial epithelial–mesenchymal transition are critical events in HNSCC progression and metastasis.

Taken together, TGM1, PPL, and KRT8 represent biologically plausible but underexplored proteins whose roles in HNSCC remain insufficiently characterized. Their involvement in epithelial integrity, cytoskeletal dynamics, and signaling pathways relevant to tumor behavior highlights the need for focused studies addressing their expression patterns and potential clinical significance in HNSCC. Our study aims to fill this apparent gap in the available literature.

It is hypothesized that the expression levels of TGM1, PPL, and KRT8 will be significantly altered in tumor and margin samples from patients with HNSCC, reflecting their potential involvement in epithelial cell structure, adhesion, and differentiation. We hypothesize that the expression levels of these proteins will be correlated with selected clinical and sociodemographic factors, which may indicate their relevance as potential biomarkers for prognosis or therapeutic targeting in HNSCC.

This study aimed to analyze the TGM1, PPL and KRT8 protein level in tumors and matched surgical margin samples from patients with HNSCC. The level of selected proteins was also evaluated in connection with clinical and demographic variables, smoking status, alcohol consumption, p16, and HPV status.

## Patients and methods

### Patients

Patients for this study were recruited from the Department of Otolaryngology and Maxillary Surgery of St. Vincent De Paul Hospital, Gdynia, Poland (Regional Medical Chamber in Gdansk, Poland no. KB-42/21 and KB-9/25). A total of 52 individuals participated in the study. The main inclusion criteria for the HNSCC group were age ≥18, diagnosis of a primary tumor and no preoperative radio/chemotherapy and signed consent to participate in the study. Details are presented in **Table 1**. The tumor and the corresponding margins were collected after surgical resection. The collected samples were histologically examined and were classified as primary HNSCC. Distant metastases were not present in any of the patients. Marginal samples were histologically confirmed to be cancer-free and were taken from the surgical margin at least 10 mm from the tumor margin. The classification and staging of the tumor specimens were in accordance with the 8th edition of the AJCC cancer staging manual (17). The clinical characteristics of the study group are presented in **Table 2**. All samples were secured and transported on dry ice to the Department of Medical and Molecular Biology, Faculty of Medical Science in Zabrze, Medical University of Silesia, Katowice, Poland, and stored at -80°C until further analysis.

**Table 1:** Characteristics of the study participants.

Variable	Category	HNSCC group N=52
Age	Years	64.3 ± 9.7
Sex	Male	36
	Female	16
Smoking	Yes	37
	No	15
Drinking	Yes - occasionally	40
	Yes - regularly	10
	No	2
Concomitant diseases	Yes	36
	No	16
HPV-DNA status	Yes	11
	No	24
	NA*	17
P16 status	Yes	8
	No	40
	NA*	4

*NA, not assessed.

**Table 2:** Clinical data of the HNSCC group.

Parameter	N (%)
HNSCC subtype	
Oral squamous cell carcinoma (OSCC)	28 (53.85)
Oropharyngeal squamous cell carcinoma (OPSCC)	1 (1.92)
Laryngeal squamous cell carcinoma (LSCC)	14 (26.92)
Hypopharyngeal squamous cell carcinoma (HPSCC)	7 (13.46)
Nasal cavity squamous cell carcinoma (NCSCC)	1 (1.92)
Skin squamous cell carcinoma (SSCC)	1 (1.92)
T classification	
T1	2 (3.85)
T2	8 (15.38)
T3	17 (32.69)
T4	25 (48.08)
Nodal status (N)	
N0	26 (50.00)
N1	9 (17.31)
N2	14 (26.92)
N3	3 (5.77)
Histological grading (G)	
G1	15 (28.85)
G2	27 (51.92)
G3	6 (11.54)
G4	1 (1.92)
NA*	3 (5.77)

*NA, not assessed.

### Experimental procedures

Tumor and corresponding surgical margin samples were weighed and mechanically homogenized in nine volumes of phosphate-buffered saline (PBS; Eurx, Gdańsk, Poland) using a PRO 200 homogenizer (PRO Scientific Inc., Oxford, CT, USA) operating at 10,000 rpm for approximately 30–40 seconds, with the tubes maintained on ice to prevent protein degradation. The resulting homogenates were subsequently subjected to sonication with a UP100H ultrasonic cell disruptor (Hielscher Ultrasonics GmbH, Teltow, Germany) at 100% amplitude, applying three 10-second pulses separated by 20-second intervals, while keeping the samples on ice to control temperature during processing. Total protein concentration was assessed with the AccuOrange™ Protein Quantitation Kit (Biotium, Fremont, CA, USA). Fluorescence intensity was recorded at an excitation wavelength of 480 nm and an emission of 598 nm using a SYNERGY H1 microplate reader (BIOTEK, Winooski, VT, USA) and Gen5 2.06 software.

The concentrations of TGM1, PPL, and KRT8 proteins were assessed by ELISA in tissue homogenates, following the procedures described by the manufacturer’s guidelines outlined in the technical manuals provided with the kits. To determine the concentrations of the tested samples, a standard curve was prepared using the standards from the kits. All standards and tissue homogenates were run in duplicates; additionally, samples for PPL were diluted 5× in phosphate-buffered saline (PBS; Eurx, Gdańsk, Poland). For all ELISA tests, plates were read by SYNERGY H1 microplate reader (BIOTEK, Winooski, VT, USA) using 450 nm as the primary wavelength. Data analysis software Gen5 2.06 was used. The concentration of the measured proteins was normalized to the total protein concentration and reported in pg/µg for all three proteins. The following tests were used: Cloud-Clone Corp., Katy, TX, USA; assay ID: SEH334Hu for PPL (with a sensitivity of 0.122 ng/mL), SEB773Hu for TGM1 (with a sensitivity of 0.121 ng/mL) and SEC025Hu for KRT8 (with a sensitivity of 0.061 ng/mL). The intra-assay variation was below 10% and the inter-assays were below 12% for the evaluated proteins.

p16 and Ki-67 was assessed by immunohistochemical staining and evaluated in the Department of Pathomorphology, Gdynia Center of Oncology, Pomeranian Hospitals. The p16 status was assessed according to widely accepted criteria used in HPV-related head and neck cancers: tumors were considered p16-positive when strong and diffuse nuclear and cytoplasmic staining was present in ≥70% of tumor cells. Cases not meeting this threshold were classified as p16-negative. DNA-HPV confirmation was performed using a PCR and “Flow-through”hybridization with GeneFlow™ HPV Array Test Kit (DiagCor Bioscience Ltd., Kowloon Bay, Hongkong) with FTPRO Flow-through System (DiagCor Bioscience Ltd., Kowloon Bay, Hongkong) and FTPRO Auto System (DiagCor Bioscience Ltd., Kowloon Bay, Hongkong) in the laboratory of the Department of Medical and Molecular Biology, Faculty of Medical Sciences in Zabrze, Medical University of Silesia in Katowice, Poland in accordance with the methodology described in our previous study (18). The extracted DNA served as a template for PCR amplification carried out on a Mastercycler Personal Thermal Cycler (Eppendorf, Hamburg, Germany). The resulting amplicons were then subjected to denaturation and hybridization, followed by enzymatic conjugation and color-development procedures. Final readouts were acquired using the FTPRO Auto System (DiagCor Bioscience Ltd., Kowloon Bay, Hong Kong). Each assay run included both manufacturer-supplied positive and negative controls to ensure result validity. In the study, we analyzed 33 HPV subtypes, including high-risk subtypes (16, 18, 31, 33, 35, 39, 45, 51, 52, 53, 56, 58, 59, 66, 68, 73, 82) and low-risk subtypes (6, 11, 26, 40, 42, 43, 44, 54, 55, 57, 61, 70, 71, 72, 81, 84).

### Statistical analysis

**Figure 1:**
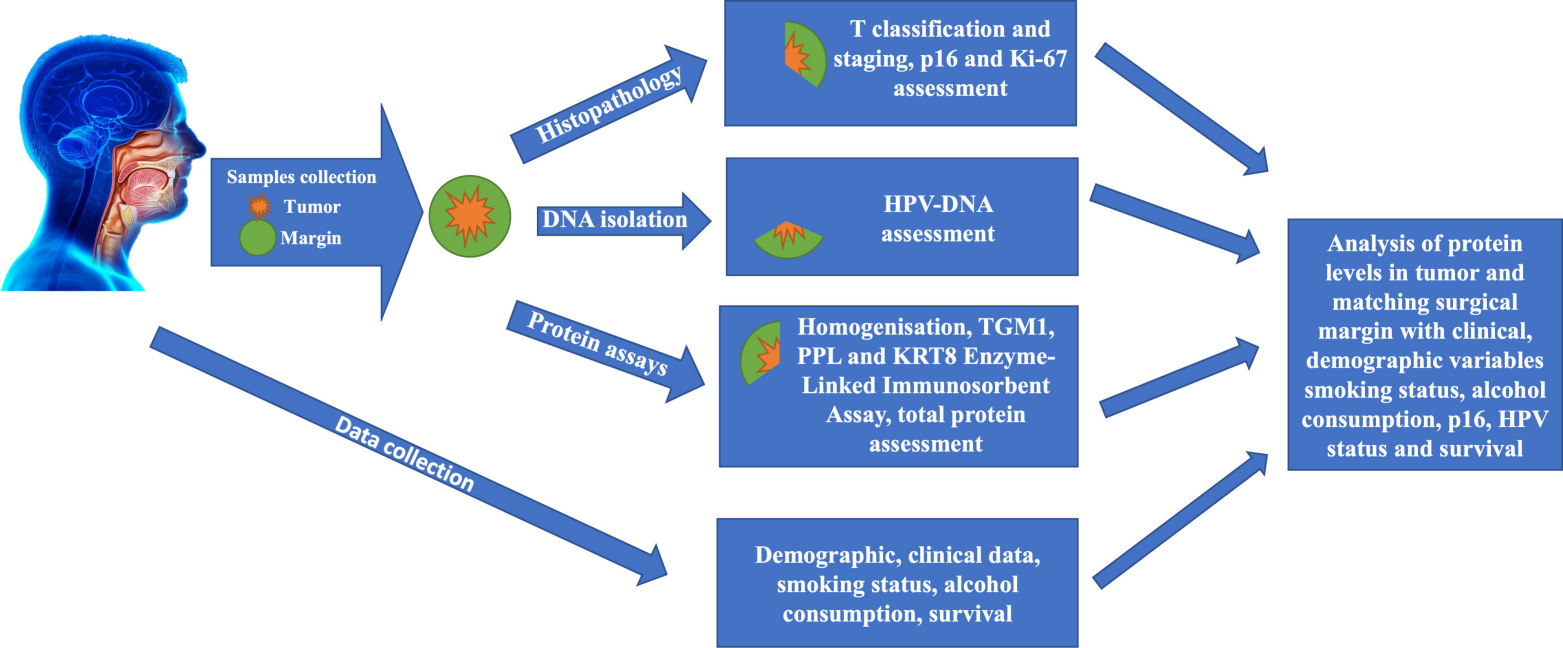
General study design.

The results were evaluated for normal distribution with the Shapiro-Wilk test. Student’s t-test, U Mann-Whitney test or Kruskal Wallis with Dun-Sidak *post hoc* were used to verify significance of differences in the means or medians between the groups. Correlation was established with Spearman’s rank correlation coefficient. For survival analyses, we used the Kaplan – Meier method with Log-Rank test where patients were divided into two groups according to protein concentration as follows: one group had protein concentration lower than median concentration and other group had protein concentration above or equal to the median concentration. Survival analysis was done for 1- and 2-years post-surgery with survival rate on the end of 1- or 2 years post surgery. Results with a *p*-value <0.05 were considered significant. STATISTICA version 13 software (TIBCO Software Inc., Palo Alto, CA, USA) was used to perform all the analyses. To avoid small groups of less than 6 cases, we combined some of them with their neighbor group as in case of T classification. But in some cases, this was impossible to achieve as in the abstinent group case. Results in the text and in the tables are presented as median with interquartile range (M (Q1-Q3)) or mean ± standard deviation (M ± SD). Survival rate is presented as ratio alive patients to all patients at the end of 1^st^ or 2^nd^ year post surgery. The study design is shown in the **Figure 1**.

## Results

### Proteins level, clinical parameters and localization of primary tumor

**Figure 2:**
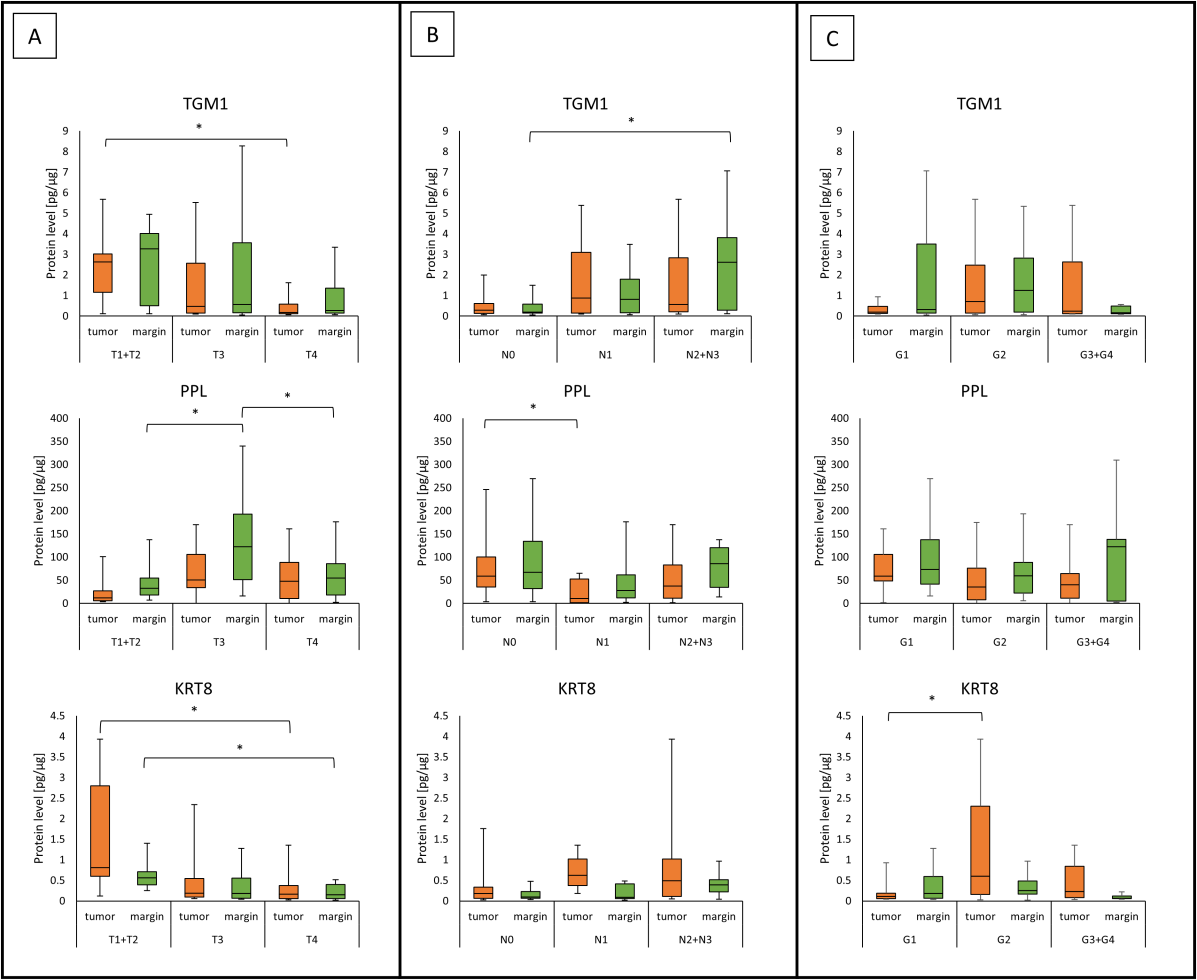
The level of TGM1, PPL, and KRT8 proteins in tumor and margin samples according to **(A)** the patient’s T status; **(B)** the patient’s N status; **(C)** tumor status G. *p<0.05.

No significant differences were found in the levels of TGM1 (0.46 (0.15 – 2.40) *vs*. 0.53 (0.15 – 2.61); *p* = 0.8419), PPL (44.28 (14.91 – 88.73) *vs*. 60.84 (22.53 – 120.25); *p* = 0.1194) and KRT8 (0.19 (0.07 – 0.59) *vs*. 0.22 (0.07 – 0.47); *p* = 0.7054) in HNSCC tumor samples compared to the margin samples. There was a higher level of TGM1 protein in tumor samples of patients with a lower T status, T1+T2 *vs*. T4 (2.63 (1.16 - 3.01) *vs*. 0.17 (0.12 - 0.58); *p* = 0.0162). PPL concentration in the margin samples was lower in the patients with clinical stage T1+T2 than in patients with T3 (32.56 (18.40 - 54.83) *vs*. 122.71 (51.43 - 193.15); *p* = 0.0276) and between patients with clinical stage T4 than in patients with T3 (54.63 (18.42 - 86.02) *vs*. 122.71 (51.43 - 193.15); *p* = 0.0247). Moreover, patients with status T1+T2 showed significantly higher levels of KRT8 protein in the tumor samples than patients with T4 status (0.81 (0.60 - 2.80) *vs*. 0.17 (0.05 - 0.37); *p* = 0.0088). Similarly, KRT8 concentration was significantly higher in the group of patients with T1+T2 status than in patients with T4 status in margin (0.56 (0.40 - 0.71) *vs*.; *p* = 0.033). The results are presented in **Figure 2A**. The group with T1 and T2 status were combined due to the small number of T1 cases.

We observed higher levels of TGM1 protein in margin samples of patients with higher nodal status, N0 *vs* N2+N3 (0.18 (0.14 - 0.58) *vs*. 2.61 (0.278-3.81); *p* = 0.0049). The higher concentrations of PPL in tumor samples showed a group of patients with N0 nodal status, which was statistically significant, N0 *vs*. N1 (58.69 (35.09 - 100.39) *vs*. 10.29 (1.55 - 52.93); *p* = 0.0356). These results are shown in **Figure 2B**. The group with N2 and N3 status was combined due to the small number of N3 cases.

Tumors with G1 had lower concentration of KRT8 protein than G2 tumors (0.11 (0.06 – 0.19) *vs*. 0.60 (0.16 – 2.31); *p* = 0.0415). No other connections were observed between protein concentration with G status in tumor and margin samples.

**Figure 3:**
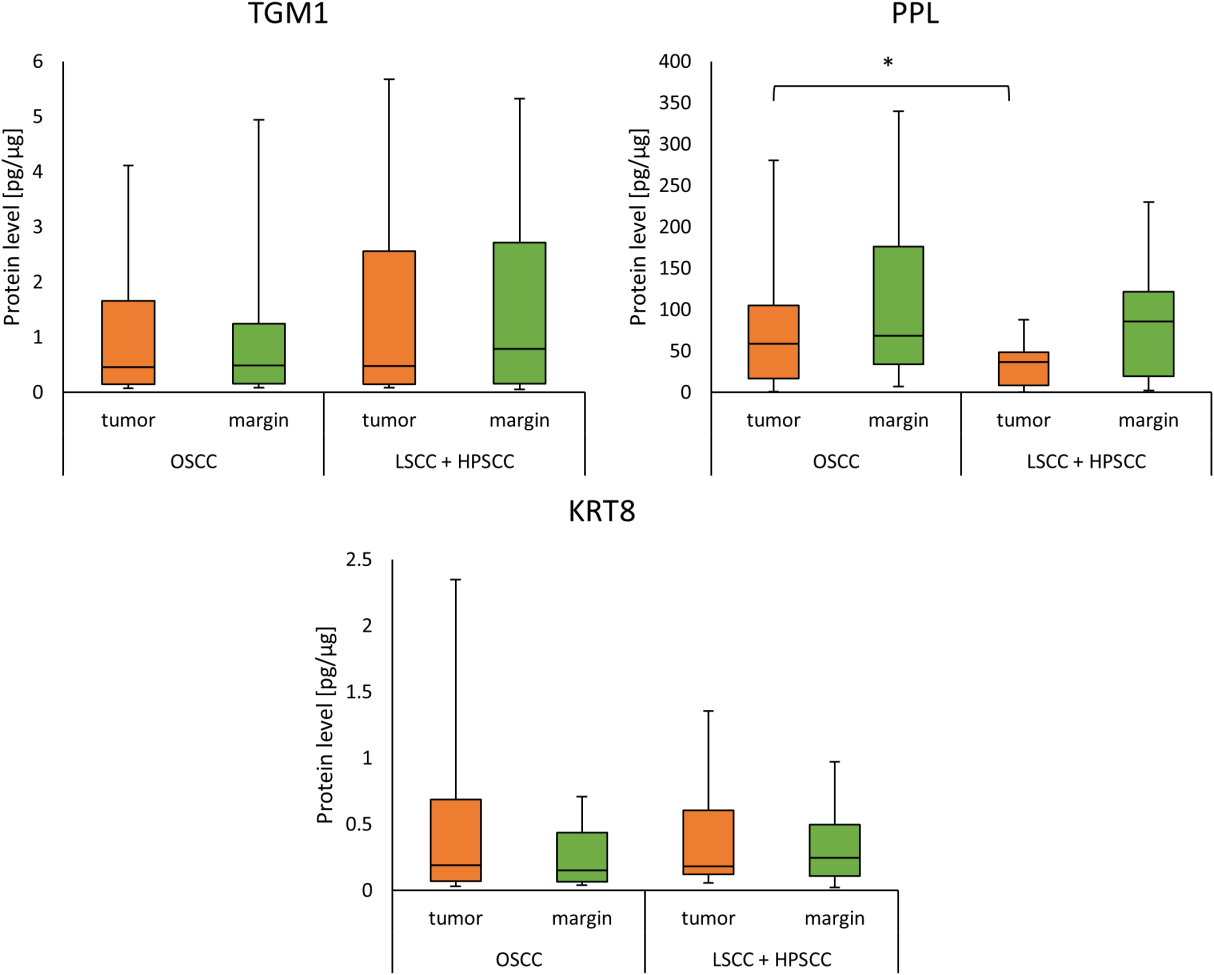
The level of TGM1, PPL, and KRT8 proteins in the tumor and margin samples according to the localization of tumor. *p<0.05.

When classifying cancers according to location, only two subgroups were studied: OSCC (28 samples) and the combined group composed of the LSCC and HPSCC subtypes (21 samples). Significant differences in PPL concentration were observed between OSCC and LSCC+HPSCC in tumor (58.69 (16.83 - 105.48) *vs*. 37.00 (8.15 - 48.39); *p* = 0.0429). The results are presented in **Figure 3**.

### Correlation of analyzed proteins in a group of patients with concomitant diseases

**Figure 4:**
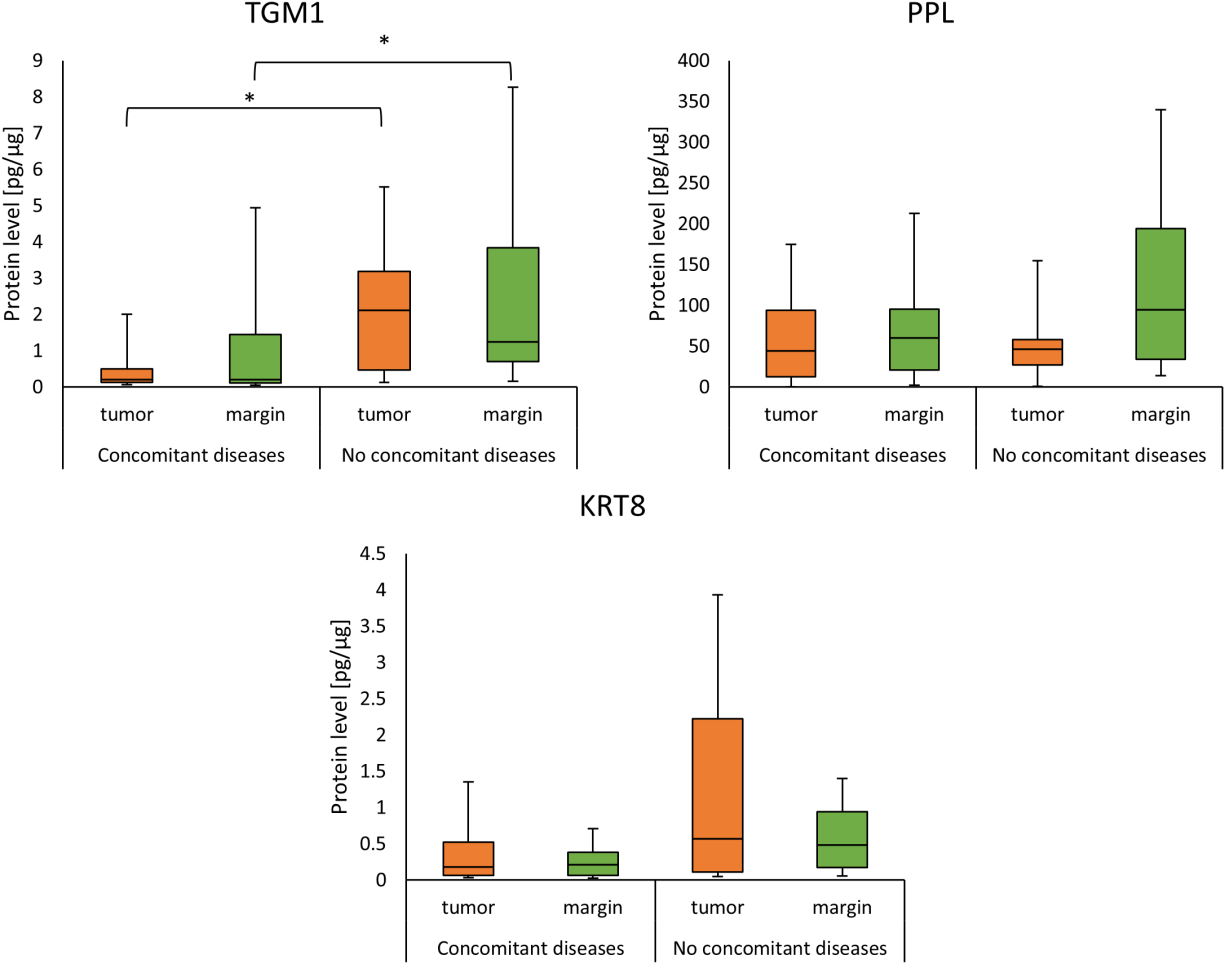
The level of TGM1, PPL, and KRT8 proteins in tumor and margin samples according to the presence of concomitant diseases. *p<0.05.

We found that the TGM1 concentration was significantly lower in tumor and margin samples in patients with any concomitant disease (cardiovascular, endocrine, digestive system or kidney diseases), compared to patients without concomitant diseases (0.21 (0.12-0.50) *vs* 2.12 (0.46 - 3.19); p=0.0010)(0.20 (0.12 - 1.45) *vs*. 1.25 (0.70 - 3.84); *p* = 0.0027), as presented in **Figure 4**.

### Concentration of analyzed proteins and Ki-67, p16, and HPV status

No significant difference in protein concentration was observed in Ki-67 positive and negative groups.

**Figure 5:**
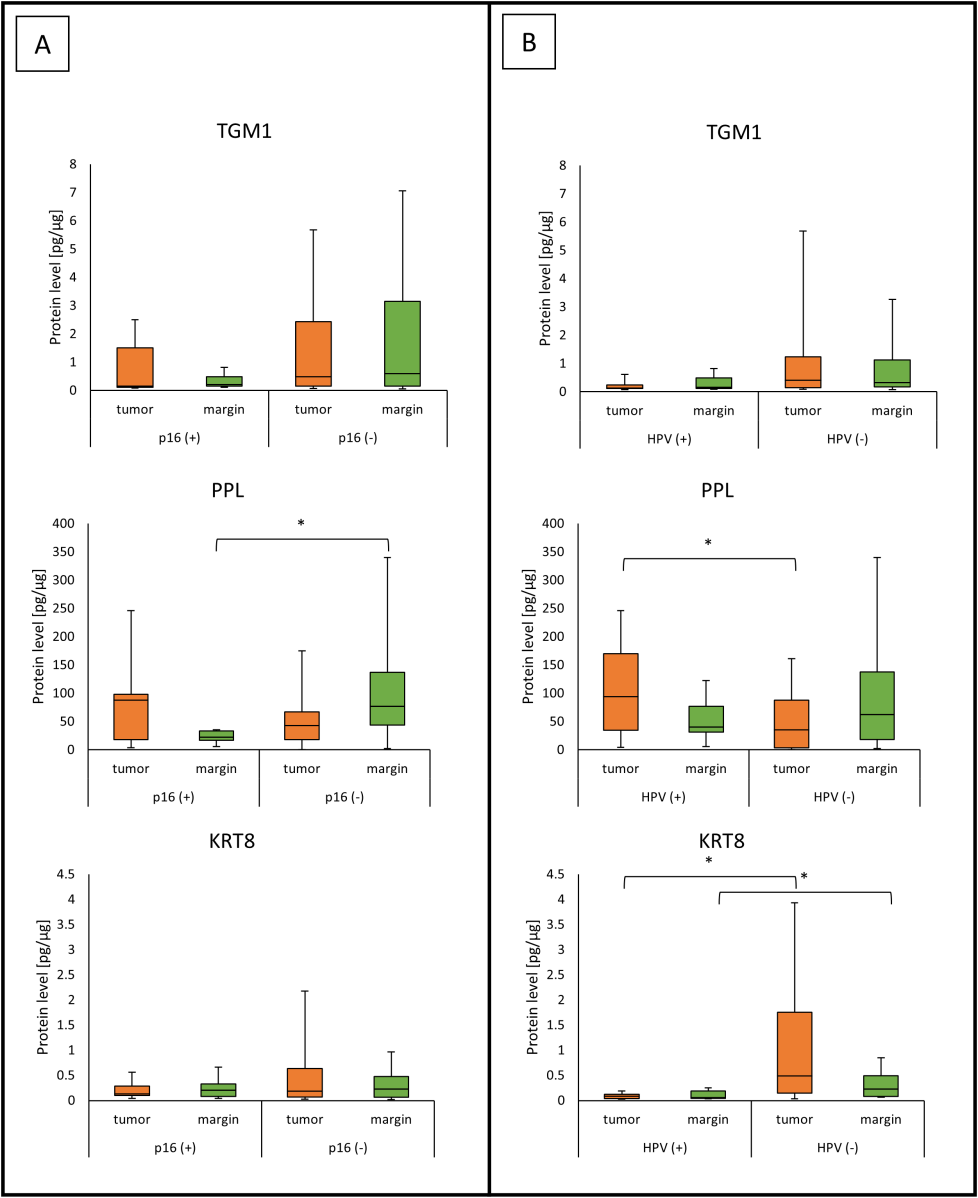
The level of TGM1, PPL and KRT8 proteins in tumor and margin samples according to the status of **(A)** p16; **(B)** HPV. *p<0.05.

Patients with p16 positive status (p16(+)) had a lower PPL concentration than patients with p16 negative status (p16(-)) in margin samples (22.64 (16.88 - 33.57) *vs*. 76.85 (43.57 - 137.23); *p* = 0.0051) as presented in **Figure 5A**.

The tumor samples from HPV-positive (HPV(+)) patients showed a higher level of PPL compared to those with negative HPV status (HPV(-)); (93.96 (34.48 - 169.78) *vs*. 35.15 (3.66 - 87.60); *p* = 0.0401). Tumor and margin samples from HPV(-) patients showed a higher level of KRT8 compared to patients with HPV(+) status; (0.49 (0.15 - 1.76) *vs*. 0.08 (0.04 - 0.12); *p* = 0.0124) (0.23 (0.08 - 0.49) *vs*. 0.06 (0.04 - 0.19); *p* = 0.0289) (**Figure 5B**). Five of our patients were both p16(+) and HPV(+).

### Concentration of analyzed proteins and alcohol and tobacco use

**Figure 6:**
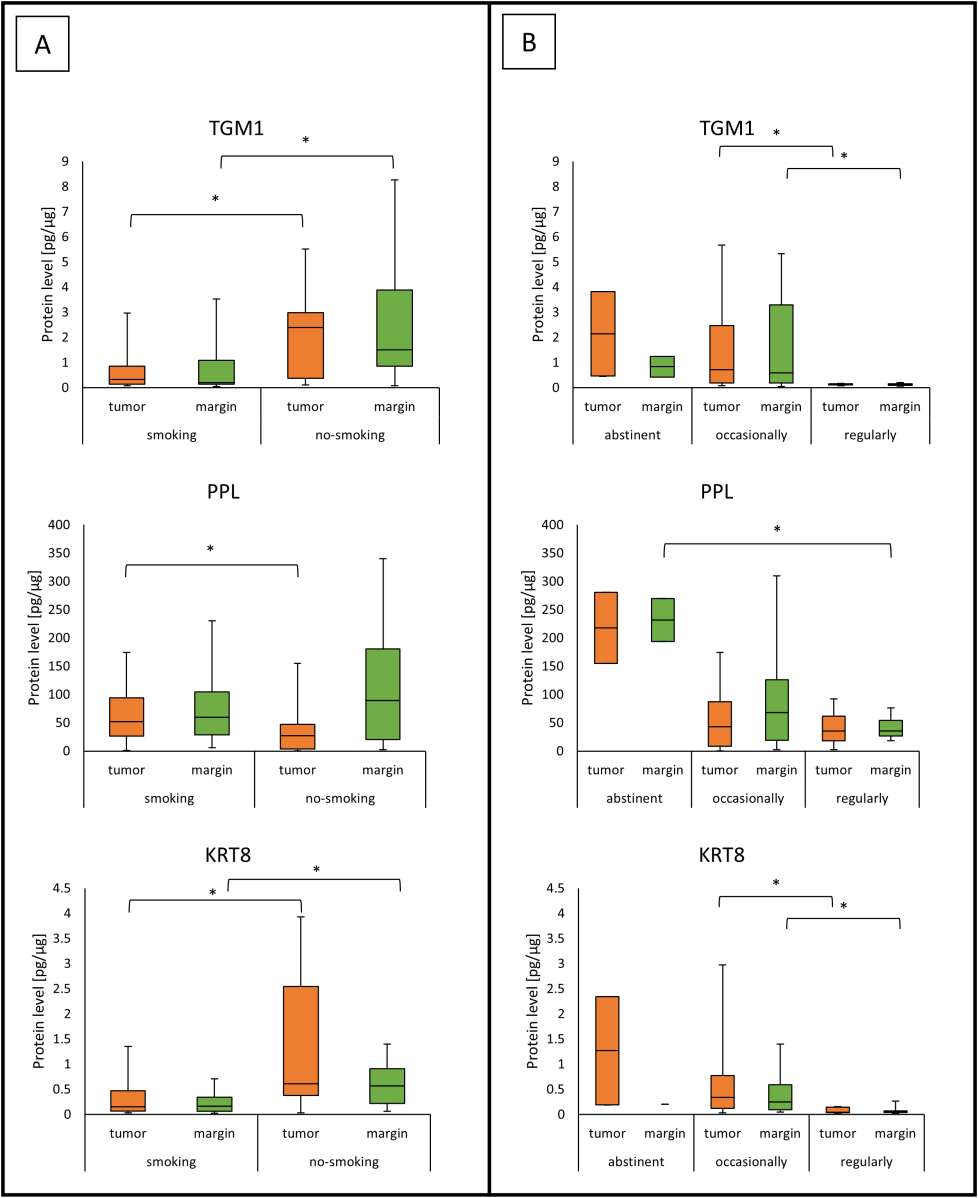
The levels of TGM1, PPL, and KRT8 proteins in tumor and margin samples according to **(A)** smoking status; **(B)** alcohol drinking status. *p<0.05.

Results for protein concentrations and alcohol or tobacco use are presented in **Figure 6**. Exact values are presented in **Table 3** with significant results in bold. For the TGM1 and KRT8 proteins, a higher level was observed in patients who drink occasionally than in those who drink regularly in tumor and margin samples (**Figure 6B**, **Table 3**). In case of smoking, TGM1 and KRT8 were higher in smokers than in patients who don’t smoke in both tumor and margin samples (**Figure 6A**, **Table 3**). PPL protein was higher in the abstinent group than in patients who regularly drink in margin samples (**Figure 6B**, **Table 3**), but it had a lower level in smokers than in non-smokers in tumor samples (**Figure 6A**, **Table 3**).

**Table 3:** The median TGM1, PPL, and KRT8 protein level and *p*-value according to smoking and drinking status.

Parameter		Smoking *vs*. no smoking	Abstinent *vs*. occasional drinker	Abstinent *vs*. regular drinker	Occasional *vs*. regular drinker	Concentration in samples [median (interquartile range)]				
						Abstinent	Occasional drinker	Regular drinker	Smoking	No smoking
TGM1	Tumor	**p= 0.0318**	p= 0.9000	p= 0.0718	**p= 0.0023**	2.14 (0.46-3.82)	0.83 (0.20-2.56)	0.12 (0.10-0.15)	0.32 (0.13-0.86)	2.39 (0.38-2.98)
	Margin	**p= 0.0003**	p= 0.9996	p= 0.2214	**p= 0.0025**	0.83 (0.41-1.25)	0.71 (0.19-3.27)	0.11 (0.10-0.16)	0.20 (0.14-1.08)	1.50 (0.86-3.88)
PPL	Tumor	**p= 0.0323**	p= 0.0907	p= 0.0880	p= 0.9788	217.90 (155.01- 280.79)	44.28 (9.22-82.67)	35.74 (18.11-62.03)	52.22 (26.80-93.96)	27.12 (3.62-46.92)
	Margin	p= 0.3932	p= 0.1286	**p= 0.0376**	p= 0.5324	231.80 (193.88-269.71)	73.43 (20.01-124.58)	35.61 (26.72-54.63)	59.78 (28.98-104.45)	89.47 (20.52-180.88)
KRT8	Tumor	**p= 0.0065**	p= 0.7865	p= 0.0894	**p= 0.0411**	1.27 (0.19-2.35)	0.34 (0.12-0.67)	0.05 (0.04-0.15)	0.15 (0.07-0.47)	0.61 (0.38-2.55)
	Margin	**p= 0.0197**	p= 0.9429	p= 0.8077	**p= 0.0049**	0.20	0.25 (0.10-0.55)	0.05 (0.04-0.07)	0.17 (0.07-0.35)	0.57 (0.22-0.91)

Bold values indicate statisticaly significant results.

### Survival analysis

We were able to obtain follow-up information for 43 of the 52 patients. Complete information was available for one year after tumor removal and half of the patients were operated upon during the last two years. The group was divided according to protein concentration into 2 groups: one group had protein concentrations lower than the median and other group had protein concentrations above or equal to the median concentration. The division was done for all proteins in tumors and in margins. There were no differences in survival between 1- and 2-years posttreatment (p>0.05), with mean survival rate 0.88 ± 0.04 in lower concentration groups and 0.89 ± 0.04 in higher concentration groups after 1 year and mean survival rate 0.76 ± 0.08 in lower concentration groups and 0.85 ± 0.08 in higher concentration groups after 2 years, detailed results are presented in **Table 4**.

**Table 4:** Survival rate of groups characterized with lower or higher concentration of TGM1, PPL or KRT8 proteins in tumor or margin samples.

Analyzed protein	follow-up time	sample type	Survival rate		p-value
			Lower concentration group	Higher concentration group	
TGM1	1 year	tumor	0.83	0.87	0.78199
		margin	0.85	0.86	0.96775
	2 year	tumor	0.66	0.87	0.24286
		margin	0.69	0.86	0.38775
PPL	1 year	tumor	0.85	0.90	0.64272
		margin	0.94	0.82	0.24177
	2 year	tumor	0.85	0.73	0.57347
		margin	0.77	0.77	0.58153
KRT8	1 year	tumor	0.86	0.94	0.45174
		margin	0.92	0.92	0.95388
	2 year	tumor	0.86	0.94	0.45174
		margin	0.75	0.92	0.27724

## Discussion

Head and neck squamous cell carcinoma (HNSCC) accounts for the majority of malignant tumors in this area, and its incidence is increasing globally (2). Despite advances in diagnosis and therapy, the disease is often detected at an advanced stage, resulting in a high mortality rate with a high rate. In recent years, interest in the molecular mechanisms of the development of HNSCC has increased, but effective diagnostic and prognostic markers are still lacking (5). Analysis of the expression of PPL, TGM1, and KRT8 proteins involved in maintaining epithelial cell structure and adhesion may provide new data on the mechanisms of tumor progression, as well as a potential source of useful biomarkers in HNSCC.

Our analysis showed no statistical differences in the expression levels of PPL, TGM1, and KRT8 proteins in the tumor samples as compared to the surgical margin samples. One possible explanation is the existence of the “field cancerization,” i.e. the presence of molecular changes in the surrounding tissue that are not yet visible histopathologically (19). Alternatively, these results may indicate that the proteins analyzed do not play a key role in differentiating tumor samples from margins in HNSCC, or that their importance may be more related to other aspects of disease progression. However, significantly higher levels of TGM1 protein were observed in patients with T1+T2 compared to T4 status in the tumor. In the context of cancer, research on the role of TGM1 is limited, and TGM1 is suspected to play a role in the early stages of HNSCC development with decreased expression with tumor progression. This may suggest involvement of TGM1 in maintaining epithelial differentiation, a characteristic that is lost in more advanced and aggressive forms of cancer. The decrease in TGM1 expression at higher clinical stages may also reflect a shift toward a more invasive cellular phenotype (20, 21). On the other hand, TGM1, by forming a network of cross-linked proteins, may impede tumor cell access to mechanisms that enable degradation of the extracellular matrix, which is crucial for invasion (22). Some studies suggest that TGM1 may interact with pathways involved in cell cycle control, apoptosis, and cell migration. By modulating these pathways, TGM1 can contribute to inhibiting tumor cell proliferation and increasing their susceptibility to apoptotic signals, which will slow tumor growth (8, 23).

Additionally, we found elevated levels of PPL in the margin samples of patients with T3 status, compared to those with T1+T2 status, as well as in patients with T3 status compared to T4. In the study by Li et al. (12), PPL expression was down-regulated in colon cancer and positively correlated with tumor size. However, direct studies investigating PPL in the context of primary tumor T status are limited, and therefore our findings can only be cautiously interpreted in light of proposed biological and adaptive mechanisms that accompany tumor progression (24). The observed increase in PPL levels in margin samples in patients with T3 status compared to T1+T2 may represent an adaptive cellular response to increasing microenvironmental stress, potentially associated with a partial epithelial-mesenchymal transition (EMT), during which cells strive to preserve adhesive properties (25). Conversely, the higher PPL levels in T3 compared to T4 may reflect an intermediate stage, wherein epithelial characteristics are still partially retained, while the T4 stage may be associated with a more complete EMT, accompanied by a decline in adhesion-related proteins, including PPL (26).

We observed a statistically significant association between higher expression of KRT8 in patients with T1+T2 stage compared to T4 in the margin and tumor. On the other hand, an immunohistochemical study by Fillies et al. in patients with oral squamous cell carcinoma found no correlation between KRT8 and tumor size (27). Another study by Alam et al. analyzed the significance of the loss of KRT8 phosphorylation at Ser73 and Ser431 residues in OSCC and found that high expression of KRT8 was correlated with tumor size (28). In the case of gastric cancer, no differences were found between tumor size and KRT8 expression using an immunohistochemical method (29). Our results can be explained as an effect of maintaining the characteristics of epithelial differentiation in the early stages of T1+T2, where tumor cells have higher expression of proteins typical of tissue of epithelial origin (30, 31). In the advanced T4 stage, EMT and cytoskeletal reorganization occurs, leading to a decrease in the expression of epithelial proteins, including KRT8 (32, 33).

We observed higher levels of TGM1 protein in the margin samples of patients with N0 *vs*. N2+N3 status. On the contrary, higher concentrations of PPL in tumor samples in a group of patients with lower nodal status was observed to be statistically significant (N0 *vs*. N1). Higher levels of TGM1 in the margins of patients with advanced nodal status may be due to the induction of mechanisms that strengthen the barrier surrounding the tumor in response to increasing tumor aggressiveness (22). In contrast, higher levels of PPL in tumor tissues in patients with N0 nodal status may indicate that stronger intercellular adhesion is maintained in the absence of tumor spread to the lymph nodes (24). The decrease in PPL expression in tumors from N1 patients may reflect the onset of the EMT process, facilitating invasion and metastasis (34). Such opposing dynamics of TGM1 in the margins and PPL within the tumor indicate different adaptive mechanisms of the tumor and surrounding tissues depending on the stage.

In this study, we observed lower KRT8 protein levels in the group of patients with G1 status when compared to patients with G2 status in tumors. This result may reflect the role of KRT8 in tumor progression and loss of cellular differentiation (35). The increase in KRT8 expression in G2 tumors may indicate a transition toward a more aggressive phenotype, which is consistent with reports suggesting that elevated KRT8 levels correlate with tumor progression, poor differentiation, and the ability to form metastases in colorectal, lung, and breast epithelial cancers (30, 36, 37).

Furthermore, we reported statistically higher PPL concentrations in the OSCC group compared to the combined group consisting of patients with LSCC and HPSCC. This may be due, among other things, to tissue specificity, as OSCC is derived from oral epithelial cells, which are strongly associated with the maintenance of desmosomal structures and thus have higher expression of PPL to maintain tissue integrity (25). In addition, OSCC may exhibit a higher degree of epithelial differentiation compared to cells in LSCC and HPSCC, and the specific oral environment may induce compensatory mechanisms, which is associated with higher expression of adhesion proteins, including PPL (12, 26). Different signaling pathways, as well as genetic and epigenetic modifications, may lead to specific regulation of PPL in different types of head and neck region cancers (38).

Moreover, we report a decreased concentration of TGM1 in patients with concomitant diseases (including cardiovascular diseases, kidney diseases, gastrointestinal diseases, endocrine diseases and others), compared to patients without concomitant disease. Our results may indicate a broader impact of systemic disorders on molecular pathways involved in epithelial homeostasis and cell differentiation (20). TGM1, as an enzyme involved in the processes of terminal differentiation and stabilization of epithelial cell structure, may be regulated by metabolic and hormonal factors, the balance of which is often disturbed in the course of chronic diseases (39). Reduced TGM1 expression in this group of patients may therefore reflect an altered intracellular and systemic environment that affects the tumor phenotype and its microenvironment, independent of classical inflammatory mechanisms (8).

Our study showed that in margin samples, the median protein concentrations of PPL were higher in p16 (–) patients than in the p16(+) group. Moreover, we found that in the case of HPV status, we observe a higher level of PPL proteins in HPV(+) tumor samples. In turn, a higher KRT8 protein level was noted in the tumor and margin in group of patients with HPV(-) status compared to HPV(+). The observed differences may reflect the different pathogenetic pathways of HPV-dependent and HPV-independent cancers. HPV(+) tumors, often p16(+), are characterized by activation of the E6/E7 pathway which disrupts the functions of p53 and Rb proteins, leading to changes in cell cycle, adhesion, and epithelial differentiation (40). In this context, higher PPL levels in HPV(+) tumors may be related to a compensatory mechanism for maintaining intercellular adhesion in response to virus-induced disruption of epithelial homeostasis. In contrast, lower levels of PPL in p16(+) margins may be indicative of a wider range of molecular changes in surrounding tissues, which may be the result of local viral or tumor field activity. In contrast, higher KRT8 levels in HPV(-) patients may result from activation of alternative signaling pathways typical of HPV-independent HNSCC, such as EGFR/PI3K/AKT and EMT (epithelial-mesenchymal transition), in which KRT8 may be involved as a marker of a transitional or more aggressive phenotype (41–43). KRT8, typical of monolayered or bilayer epithelia cytokeratin, may also reflect changes toward a less differentiated and more invasive phenotype in HPV(–) tumors.

In case of smoking, TGM1 and KRT8 were higher in smokers than in patients who don’t smoke in both tumor and margin samples. PPL had a lower level in smokers than in patients who are nonsmokers in tumor samples. Smoking, whose components include harmful, toxic and carcinogenic compounds, is one of the exogenous factors that influence changes in gene and protein expression that are crucial in cancer transformation (44). Smoking has been shown to lead to squamous cell metaplasia and epithelial hyperkeratosis and generate severe oxidative stress and chronic inflammation, activating the NF κB and AP-1 pathways that stimulate the transcription of the *KRT8* and *TGM1* genes (45, 46). In addition, chronic exposure to tobacco smoke toxins leads to cytoskeletal reorganization, activation of EMT, which is associated with degradation of desmosomal adhesion proteins, including PPL (47, 48).

In our study, we observed a higher level of TGM1 and KRT8 in patients who drink occasionally than in those who drink regularly in both tumor and margin samples. PPL protein was higher in the abstinent group than in patients who drink regularly in margin samples. Similarly to smoking, regular alcohol consumption leads to ongoing oxidative stress and inflammation, which can epigenetically suppress the expression of epithelial differentiation genes, including TGM1 and KRT8 (39). In occasional drinkers, exposure to ethanol is short-lived, which preserves higher levels of TGM1 and KRT8, consistent with a normal epithelial differentiation program (15). Lorand and Graham described that occasional contact of epithelial cells with alcohol can temporarily stimulate transglutaminase activity as a cytoprotective mechanism during short-term stress (22). Moreover, higher levels of PPL are maintained in nondrinkers, which may reflect preserved desmosomal integrity and cellular barrier (49). On the contrary, regular alcohol consumption can promote DNA hypermethylation in regions that encode adhesion proteins such as PPL, leading to reduced expression (50).

## Conclusions

We found no significant differences in the levels of PPL, TGM1, and KRT8 proteins between tumor tissue and surgical margins. While this pattern may be consistent with the concept of “field cancerization,” we acknowledge that this interpretation remains tentative in the absence of supporting molecular data such as mutational or methylation profiling. Alternative explanations for our results could be associated with protein stability, technical factors related to sample processing, or biological redundancy in epithelial tissues. Moreover, we showed significant correlation in selected protein concentrations according to tumor stage, nodal status, tumor location, HPV/p16 status, and alcohol and smoking habits, which may indicate the role of selected proteins in tumor processes, including differentiation, adhesion and EMT. In view of the results, PPL, TGM1, and KRT8 may reflect a complex interaction between epithelial differentiation, cell adhesion and micro- and macro-environmental factors affecting the development of HNSCC. The lack of extensive research on these proteins in HNSCC underscores the need for further studies to elucidate their precise roles. Further studies integrating genomic, epigenetic, and proteomic analyses will be necessary to clarify the mechanisms of PPL, TGM1 and KRT8 in HNSCC. Understanding these mechanisms could lead to novel therapeutic targets and improve patient outcomes. Overall, this research provides a valuable foundation for advancing the molecular biology of HNSCC.

The main limitation of the study was the small number of samples collected. Accordingly, some groups were combined for statistical analysis. In order to more accurately confirm the results of our analyses, further studies should be conducted on larger patient cohorts. In addition, it is necessary to conduct tests on cell lines and animal models, which could provide valuable information to better understand the role of these proteins in the development of HNSCC.

## Data Availability

The raw data supporting the conclusions of this article will be made available by the authors, without undue reservation.
